# Nematicidal Effect of Raphasatin from *Raphanus Sativus* Against *Meloidogyne Incognita*

**DOI:** 10.2478/jofnem-2022-0050

**Published:** 2022-11-17

**Authors:** Nadhem Aissani, Hichem Sebai

**Affiliations:** 1 Laboratory of Functional Physiology and Valorization of Bioressources, Higher Institute of Biotechnology of Beja, University of Jendouba, Jendouba Tunisia

**Keywords:** DPPH, isothiocyanates, *Meloidogyne incognita*, *Raphanus sativus*, raphasatin

## Abstract

The nematicidal activity of radish bulb (*Raphanus sativus*) methanol (RME) and aqueous extracts (RAE) was tested against the second stage (J_2_) root knot nematode *Meloidogyne incognita* model. The EC_50_ of RME after 3 d of J_2_ immersion in test solutions was 312 ± 65 μg/ml. However, no activity was noted for RAE (EC_50_ > 1,000 μg/ml). The chemical composition analysis of the methanol extract carried out by the GC–MS technique showed that 4-methylthio-3-butenyl isothiocyanate (raphasatin) was the most abundant compound at 20%. This pure compound strongly induced J_2_ paralysis with an EC_50_ of 1.3 ± 0.2 μg/ml after 24 hr. Comparison with other 11 selected isothiocyanates with structure similarity showed that the double bond at position 3 of the alkyl side chain is important for nematicidal activity, followed by the sulfur group at position 5 and the benzyl group at position 2. In addition, raphasatin showed the highest nematicidal activity with the corresponding lowest antioxidant activity of about 92 ± 18 μg/ml. In conclusion, the results of this investigation reveal that *R. sativus* and its major compound raphasatin can be integrated into the pest management system.

Root-knot nematodes (RKN) of the genus *Meloidogyne* represent a serious problem in crop production (Koenning *et al*., 1999) owing to their short biological cycle along with secondary infections caused to the host root by soil pathogens, often leading to total harvest loss. In the past, nematode control was mainly achieved by the use of synthetic fumigant nematicides such as methyl bromide, which has now been withdrawn from the market owing to degradation of the ozone layer (Caboni *et al*., 2012). Thus, plant protection from RKN and soil-borne plant pathogens should therefore rely on alternative control strategies that are both economically sustainable and environmentally sound at the same time.

Plants can produce compounds that directly or indirectly affect their biological environment. These compounds have a dramatic influence on the life cycle of the surrounding living organisms (Whittaker and Feeny, 1971). Such compounds, called allelochemicals, have nematicidal, insecticidal, hormonal, and antifeedant characteristics against pests, and additionally exhibit antimicrobial activities (Copp, 2003; Isman, 2006). Many scientific studies have reported data on the biological activity of plant secondary metabolites on RKN (Aissani *et al*., 2013, 2015). *Sambucus nigra* and *Melia azedarach* methanol extracts were reported to have nematicidal activity against *Meloidogyne incognita* (Akyazi, 2014). Aqueous extracts from sparagus, whitehibiscus, jasmine, and Ixora caused mortality of *M. incognita* juveniles in a dose–response manner (Vinodhini *et al*., 2019).

Radish (*Raphanus sativus*), belonging to the *Brassicaceae* family, is used in indigenous Asian herbal medications (Shukla *et al*., 2011) and has dietary nutritional values (Hanlon and Barnes, 2011). Radish has been reported to possess the capability for a wide range of pharmacological activities such as gut stimulatory (Gilani and Ghayur, 2004), hepatoprotective (Salah-Abbès *et al*., 2009), cardioprotective (Zaman, 2004), antioxidant (Barillari *et al*., 2006), antitumor, and anti-inflammatory activities (Kim *et al*., 2011, 2014), and these are attributed to the presence of isothiocyanates. These compounds are volatile secondary metabolites that derive from the enzymatic hydrolysis of their precursors, glucosinolates, in numerous plants belonging to the *Brassicaceae* family, by myrosinase (EC 3.2.1.147) after tissue damage (Aissani *et al*., 2013). These components give the characteristic smell and pungent flavor of these plants.

Except for the study of Aydinli and Mennan (2018) dealing with the nematicidal activity of *R. sativus* on *M. arenaria* juveniles, there are no reports on the nematicidal activity of this plant and its phytochemicals on *M. incognita*, the most destructive pathogen of many crops.

In the present investigation, we report for the first time: (i) the GC–MS chemical characterization of the bulb of *R. sativus* methanol extract (RME), (ii) the study of the in vitro biological activity (EC_50_) of RME and its major compound having a nematicidal effect against *M. incognita* second-stage juveniles (J_2_), (iii) preliminary structure–activity relationship with other 11 selected isothiocyanates, and (iv) the correlation between nematicidal analysis and antioxidant power of the tested compounds.

## Materials and Methods

### Extraction of pant materials

Fresh *R. sativus* was obtained from a local market in Beja-Tunisia (geographical coordinates: 36°43′32′′N/9°10′54′′E) in January, 2017. It was identified according to the flora of Tunisia by Professor Hichem Sebai. Bulbs were air-dried for 7 d at room temperature (20 ± 2°C), ground into a fine powder using a Retsch blender mill, sifted through a 0.5 mm mesh screen to obtain a uniform particle size, stored in the Laboratory of Functional Physiology and Valorization of Bioresources, High Institute of Biotechnology of Beja, Tunisia, with the voucher specimen number # RS-BL-20-01-17, and subsequently used for extraction.

Then, 100 g of ground bulb was extracted using 400 ml of water or methanol (HPLC grade), sonicated for 15 min, and macerated for 24 hr at room temperature. The extracts were centrifuged for 15 min at 13,000 rpm after being filtered through a Whatman No. 40 filter paper. We didn’t use a rotary evaporator to dry the extracts to avoid the loss of volatile compounds such as isothiocyanates. The extracted samples were kept at 4°C until further analysis. The plant material was also dried at 105°C for 24 h, and the moisture content was found to be 62.51%.

## GC–MS analysis

Chromatographic separation and identification of components of methanol extract of *R. sativus* were performed on a gas chromatograph Varian model 3800 (Varian, Milan, Italy) equipped with a Varian 7800 autosampler, a split/splitless injector Varian 1079, and an ion trap mass detector (ITMS) 2000. The analytical column was a Varian VF5m s (30 m × 0.25 mm i.d. × 0.25 mm film thickness). Helium was the carrier gas at 1 ml/min. Then, a 1-μl sample, previously dehydrated with sodium sulfate and filtrated, was injected in the splitless mode with a purge valve on for 1 min. The injector temperature was set at 200°C. The mass spectrometer detector was operated in the electron ionization positive mode. Trap, manifold, and transfer line temperatures were at 200°C, 80°C, and 200°C, respectively. The oven was programmed as follows: 50°C (1 min), raised to 100°C (5°C/min), held for 1 min, then raised to 180°C (20°C/min), and held for 4 min. Qualitative analysis was performed in the scan mode (50–550 amu). Peak identification was made by comparing full mass spectra and retention times from authentic standards and the NIST MS Spectra Library (The NIST Mass Spectral Search Program for the NIST\EPA\NIH mass Spectral Library, version 2.0, build 12/2000).

## Nematode population

A population of *M. incognita* originally reared from tomato roots (*Solanum lycopersicum* L.) cv. Belladonna, a cultivar that is very susceptible to RKN, was collected from a greenhouse in Cagliari (Italy). All plants were maintained in a growth chamber at 25°C to 28°C, with 60% relative humidity and a 16-h photoperiod, in plastic pots (18-cm diameter). Plants used for inoculations were 7 wk old, at the five-leaf stage. After 40 d, the plants were uprooted, and the roots were washed to be free of soil and cut into 2 cm pieces. Eggs were extracted according to the sodium hypochlorite procedure, and J_2_ were allowed to hatch in modified Baermann funnels at 28°C. All J_2_ hatching in the first 3 d were discarded, and thereafter, J_2_ collected after 24 h were used in the experiments (Aissani *et al*., 2017).

## Nematicidal assay

*Raphanus sativus* methanol extract and *Raphanus sativus* aqueous extracts (RAE) were tested against RKN at a dose range of 10−5,000 μg/ml and assessed for toxicity at 72 h using fosthiazate as a chemical control according to Aissani *et al*. (2013). Nematicidal activity of raphasatin (Sigma Aldrich, Tunis, Tunisia) found in RME was compared with that of 11 aliphatic and aromatic isothiocyanates previously tested against J_2_ at a dose ranging from 1 μg/ml to 1,000 μg/ml using the organophosphorus fosthiazate (Sigma Aldrich) as a chemical control (Aissani *et al*., 2013, 2015). Stock solutions of pure compounds were prepared in methanol to overcome insolubility, whereas Tween 20 in twice distilled water was used for further dilution. The final concentration of methanol in each well never exceeded 1% (v/v) since preliminary experiments showed that this concentration was not toxic to nematodes. In all cases, working solutions were prepared containing double the test concentration and mixed in CellstarR 96-well cell culture plates (Greiner Bio-One) at a ratio of 1:1 (v/v) with a suspension of 25 J_2_ added to each well. Multiwell plates were covered to avoid evaporation and maintained in the dark at 20°C. Juveniles were observed with the aid of an inverted microscope (Zeiss, 3951, Germany) at ×10 and ×20 after 72 h for extracts and 24 h for pure compounds. J_2_ were ranked in two distinct categories: motile or paralyzed/dead. Paralysis experiments were performed in three replicates.

## Antioxidant assay

Chemically, the more a compound is oxidant, the more it will generate free radicals and consequently the more it has nematicidal activity. The antioxidant capacity of the tested compounds was performed using 2,2-diphenyl-1-picrylhydrazyl (DPPH) radical-scavenging activity, as described previously by Grzegorczyk *et al*. (2007). Briefly, various concentrations of phenols (0.3–200 μg/ml) were added to 1 ml of 0.1-mM methanol solution of DPPH and incubated at 27°C for 30 min. The optical density of the sample was quantified at 517 nm. DPPH radical-scavenging activity (RSA), expressed as percentage, was estimated utilizing the following formula:


$$\left.RSA(\text{\%})=\left[A_{DPPH}-\left(A_{\text{sample }}-A_{\text{control }}\right)/A_{DPPH}\right)\right]\times 100$$

Ascorbic acid (Sigma Aldrich) was used as a reference molecule in the same concentrations as the tested extract. All the analyses were carried out in triplicates. The efficacy concentration 50 (EC_50_) value was determined as the concentration of the compound required to scavenge 50% of the DPPH radicals.

## Statistical analysis

Treatments of the paralysis experiments were replicated thrice, and each experiment was performed twice. The percentages of paralyzed J_2_ in the microwell assays were corrected by removal of the natural death/paralysis percentage in the water control, according to the Schneider Orelli formula, corrected (Puntener, 1981):


$$\begin{array}{rl} & \text{\%}=[(\text{ paralysis \% in treatment }-\text{ paralysis \% in }\\ & \text{ control)/(100 - paralysis \% in control) }]\times 100 \end{array}$$

and they were analyzed by ANOVA. Since it showed no significant treatment by time interaction, means were averaged over experiments. Corrected percentages of paralyzed J_2_ treated with test compounds were subjected to non-linear regression analysis using the log-logistic equation proposed by Seefeldt *et al*. (1995):


$$\left.\left.y=C+(D-C)/\left\{1+\exp\left[b\log(x)-\log{EC}_{50}\right)\right)\right]\right\}$$

where C is the lower limit, D is the upper limit, b is the slope at the EC_50_, and EC_50_ is the test compound concentration required for 50% paralyzed helminths after control elimination (natural death/paralysis). In the regression equation, the test compound concentration (% w/v) was the independent variable (x), and the paralyzed J_2_ was the dependent variable (*y*). The mean value of the six replicates per compound concentration and immersion period was used to calculate the EC_50_ value.

## Results

### Nematicidal activity of extracts

**Figure 1:**
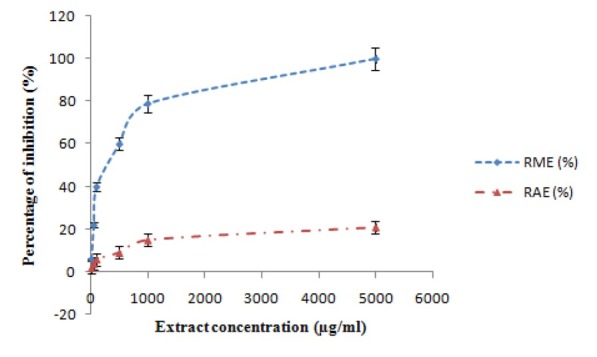
Dose–response relationship between extract concentration and percentage of J_2_ inhibition. RAE, *Raphanus sativus* aqueous extracts; RME, *Raphanus sativus* methanol extract.

When RME was tested against *M. incognita*, a clear dose−response relationship was established and significant paralysis/death of J_2_ was evident after 3 d of exposure with a calculated EC_50/3 d_ value of 312 ± 65 μg/ml (Fig. 1). However, no activity was recorded for RAE.

### GC–MS analysis of RME

Using GC–MS, we were able to identify and quantify the most abundant metabolite of methanol extract, 4-methylthio-3-butenyl isothiocyanate (raphasatin), at 20%, while the identification, by the NIST library of other constituents of the extract, was unsuccessful.

### Nematicidal activity of pure compounds

Compared with the selected isothiocyanates previously tested against J_2_, 4-methylthio-3-butenyl was the most active followed by benzyliosothiocyante, 4-methylthiobutyl isothiocyanate (erucin), allylisothiocynate, and methylisothiocynate with an EC_50/1 d_ of 1.3 ± 0.2 μg/ml, 1.9 ± 0.3 μg/ml, 3.2 ± 1.7 μg/ml, 6.6 ± 2.5 μg/ml, and 7.9 ± 3.1 μg/ml, respectively (Table 1).

**Table 1:** Correlation between nematicidal and antioxidant activity of the tested compounds (*n* = 3).

Compound	MW (g/mol)	EC_50/24 h_ (μg/ml)	EC_50_ DPPH (μg/ml)
Raphasatin	159.27	1.3 ± 0.2	92 ± 18
Benzylisothiocyanate	149.21	1.9 ± 0.3^a^	90 ± 17
Erucin	165.21	3.2 ± 1.7^a^	43 ± 12
Allylisothiocyanate	99.15	6.6 ± 2.5^a^	32 ± 4.3
Methylisothiocyanate	73.12	7.9 ± 3.1^a^	20 ± 2.6
Pentylisothiocyanate	129.23	11.1 ± 5.0^a^	9.4 ± 1.7
Hexylisothiocyanate	127.18	11.3 ± 2.6^a^	nt
Butylisothiocyanate^c^	115.20	12 ± 8.0^a^	9.1 ± 1.5
Sulforaphane	177.3	152 ± 35^a^	nt
Iberin	163.3	180 ± 21^a^	6.55 ± 1.1
Isobutylisothiocyanate	115.20	>200^a^	5.3 ± 0.9
Phenylisothiocyanate	135.19	>1,000^a^	2.6 ± 0.7
Fosthiazate	283.35	0.4 ± 0.3^a^	nt
Ascorbic acid	170.12	nt	1.15 ± 0.9^b^

EC_50/24 h_: Half maximal effective concentration of the tested compounds on J_2_ after 24 hr EC_50_DPPH: Half maximal effective concentration of the tested compounds to scavenge DPPH ^a^Data reported by Aissani *et al*. (2015). ^b^Data reported by Aissani *et al*. (2017). ^c^This compound has a fumigant activity. MW, molecular weight; nt, not tested.

### Antioxidant assay

The DPPH radical-scavenging activity method showed that the most antioxidant compound was phenylisothiocynate **(12)** followed by isobutylisothiocyanate **(11)** and 3-methylsulfinylpropyl isothiocyanate (iberin) **(10)**, with EC_50/24 h_ values of 2.6 ± 0.7 μg/ml, 5.3 ± 0.9 μg/ml, and 6.55 ± 1.1 μg/ml, respectively. The least antioxidant compound was raphasatin **(1)** followed by benzylisothiocyanate **(2)**, and erucin **(3)**, with EC_50_ values of 92 ± 18 μg/ml, 90 ± 17 μg/ ml, and 43 ± 12 μg/ml, respectively (Table 1).

## Discussion

Only methanol extract showed a nematicidal activity against *M. incognita* with an EC_50/3 d_ value of 312 ± 65 μg/ml. Aqueous extract containing five phenol compounds according to our recent study was not active against J_2_ (Aissani and Sebai, 2022). This result is not in agreement with that of Aoudia *et al*. (2012) dealing with nematicidal activity of *M. azedarach* against *M. incognita*. This can be due probably to the difference of chemical composition of the two plants. GC–MS analysis allowed us to identify the most abundant compound, 4-methylthio-3-butenyl isothiocyanate, at 20%. Our results are similar to those found by Salah-Abbès *et al*. (2009) using the same Tunisian variety Melito of *R. sativus*. However, Beevi *et al*. found several isothiocyanates in the hexan extract of Indian radish roots, including sulforaphene (Beevi *et al*., 2010). This confirms that extraction yield is influenced by the origin of plants, solvent used for extraction, and analysis method. To the best of our knowledge and according to literature survey, this compound was found only in radish.

4-Methylthio-3-butenyl isothiocyanate applied against J_2_ was strongly active with an EC_50_ of 1.3 ± 0.2 μg/ml after 24 hr. This is the first report of the nematicidal activity of raphasatin as a constituent of *R. sativus*.

Compared with other isothiocyanates with structure similarities, we noted that slight structural differences can confer important different nematicidal effects, confirming that biological activity is a function not only of the concentration of the product but also of the chemical properties of the alkyl side chain. Addition of the double bond at position 3 increased the nematicidal activity more than twice from an EC_50/24 h_ of 3.2 ± 1.7 μg/ml to 1.3 ± 0.2 μg/ml. This is the case observed with each erucin **(3)** and raphasatin **(1)**. Besides, the addition of sulfur group at position 5 of the side alkyl chain increased the nematicidal activity more than thrice in the case of erucin **(3)** and hexylisothiocyanate **(7)**. Moreover, when the double bond in position 2 was substituted by a benzene moiety as in the case of allyisothiocyanate **(4)** benzyl isothiocyanate **(2)**, the compound increased thrice from an EC_50/24 h_ of 6.6 ± 3.4 μg/ml to 1.9 ± 1.1 μg/ml (Table 1) (data not shown).

**Figure 2:**
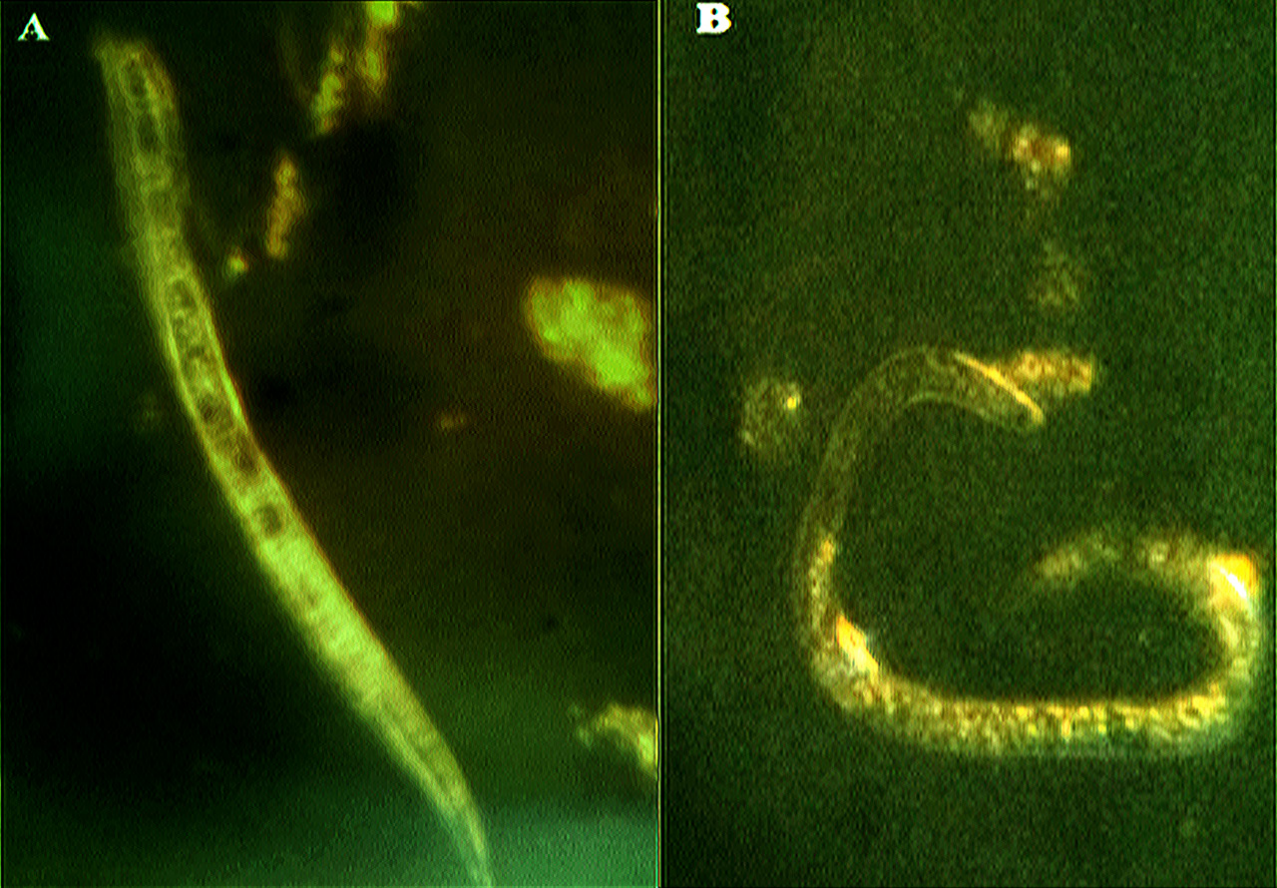
Nematodes treated by 5 μg/ml of raphasatin (A) or fosthiazate (B). Nematodes were paralyzed in a straight shape, and internal vacuoles were evident in (A) while in (B) they were paralyzed in a coiling shape.

Remarkably, raphasatin showed the highest nematicidal activity with the corresponding lowest antioxidant activity (about 97 ± 20 μg/ml) (Table 1). Isothiocyanates are not able to scavenge free radicals such as the superoxide anion (O2) and hydroxyl radical (OH), and their toxicity is revealed by the production of reactive oxygen species (ROS). The isothiocyanate treatments paralyzed nematodes in a straight shape with evident internal vacuolization (Aissani *et al*., 2013). Conversely, nematodes treated with the organophosphorous fosthiazate were paralyzed in a coiling shape (Aissani *et al*., 2015) (Fig. 2). Isothiocyanates are generally biocides and highly electrophile with the capability of reacting with sulfhydryl groups and binding to cysteine-altering protein functions. Previous studies demonstrated that isothiocyanates and aldehydes blocked nematode V-ATPase (Aissani *et al*., 2013, 2015). In the same fashion, gossypol and phenols were shown to inhibit mitochondrial electron transfer and stimulate generation of ROS (Aissani *et al*., 2017). Thus, ROS produced by isothiocyanates may have a crucial role in the inhibition of V-ATPase and induction of lipid peroxidation, as well as depletion of antioxidant enzyme activities, such as superoxide dismutase, catalase, and glutathione peroxidase (Aissani *et al*., 2017).

A previous study reported the depletion of monoacylglycerol levels when *M. incognita* was treated with V-ATPase inhibitors such as maleimide and arylhydrazones (Eloh *et al*., 2016). Moreover, 5-oxoproline, a derivative of γ-glutamyl amino acid, was a downregulated discriminant in treated nematodes. It is known that γ-glutamyl amino acid is formed from glutathione normally transported out of cells where transpeptidation occurs in the presence of amino acids (Anderson, 1996). We speculated that pyroglutamic acid diminution might be promoted by glutathione depletion.

This article deals with the study of the nematicidal activity of the radish methanol extract against *M. incognita*. Radish is rich in 4-methylthio-3-butenyl isothiocyanate, which has nematicidal activity against this RKN and allows us to use this plant for crop protection against pests. Comparison with other isothiocyanates showed that double bounds at position 3 are very important for nematicidal activity. This gives insights that would enable the development of new pesticides with high nematicidal activity.

## Supplementary Material

4-methylthio-3-butenyl isothiocyanate (raphasatin) **(1)**

4-methylthiobutyl isothiocyanate (erucin) **(3)**

methylisothiocyanate **(5)**

hexylisothiocyanate **(7)**

4-(methylsulfinyl) butylisothiocyanate (sulforaphane) **(9)**

isobutylisothiocyanate **(11)**

fosthiazate **(13)** Chemical structure of the tested compounds

benzylisothiocyanate **(2)**

allylisothiocyanate **(4)**

pentylisothiocyanate **(6)**

butylisothiocyanate **(8)**

3-methylsulfinylpropyl isothiocyanate (iberin) **(10)**

phenylisothiocyanate **(1 2)**
